# Rapid, Easy, and Reliable Identification of *Nocardia* sp. by MALDI-TOF Mass Spectrometry, VITEK^®^-MS IVD V3.2 Database, Using Direct Deposit

**DOI:** 10.3390/ijms24065469

**Published:** 2023-03-13

**Authors:** Elisabeth Hodille, Clémence Prudhomme, Oana Dumitrescu, Yvonne Benito, Olivier Dauwalder, Gérard Lina

**Affiliations:** Hospices Civils de Lyon, Laboratoire de Biologie Médicale Multi-Sites, Centre de Biologie et Pathologie Nord, Institut des Agents Infectieux, Laboratoire de Biologie Médicale de Référence des Nocardioses, 69004 Lyon, France; clemence.prudhomme@etu.univ-lyon1.fr (C.P.); oana.dumitrescu@chu-lyon.fr (O.D.); yvonne.benito@chu-lyon.fr (Y.B.); olivier.dauwalder@chu-lyon.fr (O.D.); gerard.lina@chu-lyon.fr (G.L.)

**Keywords:** *Nocardia*, matrix-assisted laser desorption/ionization time-of-flight mass spectrometry, VITEK^®^-MS, VITEK^®^-PICKME^TM^ pen, direct deposit

## Abstract

The reference methods for *Nocardia* identification are based on gene sequencing. These methods are time-consuming and not accessible for all laboratories. Conversely, matrix-assisted laser desorption/ionization time-of-flight (MALDI-TOF) mass spectrometry is easy to use and widely available in clinical laboratories, but for *Nocardia* identification, the VITEK^®^-MS manufacturer recommends a tedious step of colony preparation that is difficult to integrate into a laboratory workflow. This study aimed to evaluate *Nocardia* identification by MALDI-TOF VITEK^®^-MS using direct deposit with the VITEK^®^-PICKME^TM^ pen and a formic acid-based protein extraction directly onto the bacterial smear on a 134 isolates collection; this identification was compared to the results from molecular reference methods. For 81.3% of the isolates, VITEK^®^-MS delivered an interpretable result. The overall agreement with the reference method was 78.4%. Taking only the species included in the VITEK^®^-MS in vitro diagnostic V3.2 database into account, the overall agreement was significantly higher, 93.7%. VITEK^®^-MS rarely misidentified isolates (4/134, 3%). Among the 25 isolates that produced no result with the VITEK^®^-MS, 18 were expected, as *Nocardia* species were not included in the VITEK^®^-MS V3.2 database. A rapid and reliable *Nocardia* identification using direct deposit by VITEK^®^-MS is possible by combining the use of the VITEK^®^-PICKME^TM^ pen and a formic acid-based protein extractiondirectly onto the bacterial smear.

## 1. Introduction

*Nocardia* species, responsible for nocardiosis, are filamentous, Gram-positive bacteria belonging to the order Corynebacteriales, cosmopolitan and ubiquitous in the environment. Nocardiosis is a rare infection, primarily opportunistic and mostly affecting immunocompromised patients, although immunocompetent patients can also be affected [1,2]. Nocardiosis is often difficult to treat and may be life threatening, requiring a prompt initiation of effective antibiotic therapy. Importantly, an accurate species identification offers the potential to partially predict antimicrobial susceptibility [3] and, therefore, contributes to the selection of therapy [1].

The gold standard for *Nocardia* species identification is molecular biology with amplification and sequencing of one or two gene(s) among either 16S rRNA, *hsp65*, *secA1*, or *sodA* [4,5,6,7]. However, not all laboratories are capable of these molecular biology techniques that require specific equipment; in addition, the turnaround time can be more than 24 h. In recent years, matrix-assisted laser desorption/ionization time-of-flight mass spectrometry (MALDI-TOF) has revolutionized bacterial identification in microbiology laboratories, making it possible to obtain rapid (within a few minutes), easy, inexpensive, and reliable identification from growth on agar [8].

For *Nocardia* identification using VITEK^®^-MS (MALDI-TOF) coupled with the in vitro diagnosis (IVD) database (bioMérieux, Marcy l’Etoile, France), several studies have reported good performance of the VITEK^®^-MS IVD database [9,10,11,12], but its use is hampered by a sample preparation recommended by the manufacturer. This preparations calls for the user to lyse the bacterial wall and assist in the extraction of ribosomal proteins that determine organism identification, notably, by treatment with formic acid; however, this procedure is difficult to integrate into a laboratory workflow because it is time-consuming, with several incubation and centrifugation steps. Without this sample preparation, identification yields drop to <15% after direct deposit using standard loops [10]; on the one hand, due to the difficulty of obtaining fine, good quality smears from filamentous, rough, and dry colonies embedded in the agar with a loop; and, on the other hand, due to the lack of formic acid treatment of the deposit.

MALDI-TOF users have previously reported that the use of a formic acid-based protein extraction directly onto the bacterial smear before matrix application is required in order to obtain correct identification, mainly for Gram-positive bacteria bacilli and yeast [13]. The VITEK^®^-PICKME^TM^ pen (bioMérieux) is a device that includes single-use nibs, facilitating the user’s ability to pick and smear colonies on the VITEK^®^-MS target slide to improve the quality of smearing. Compared to the loop, the pen provides better homogeneity and a thinner deposit.

Herein, we evaluated the performance of *Nocardia* identification by the VITEK^®^-MS instrument coupled with the VITEK^®^-MS IVD V3.2 database using direct deposit with the VITEK^®^-PICKME^TM^ pen and a formic acid-based protein extraction directly onto the bacterial smear. This simplified protocol could be easily integrated into a laboratory workflow.

## 2. Results

### 2.1. Nocardia Collection

**Figure 1:**
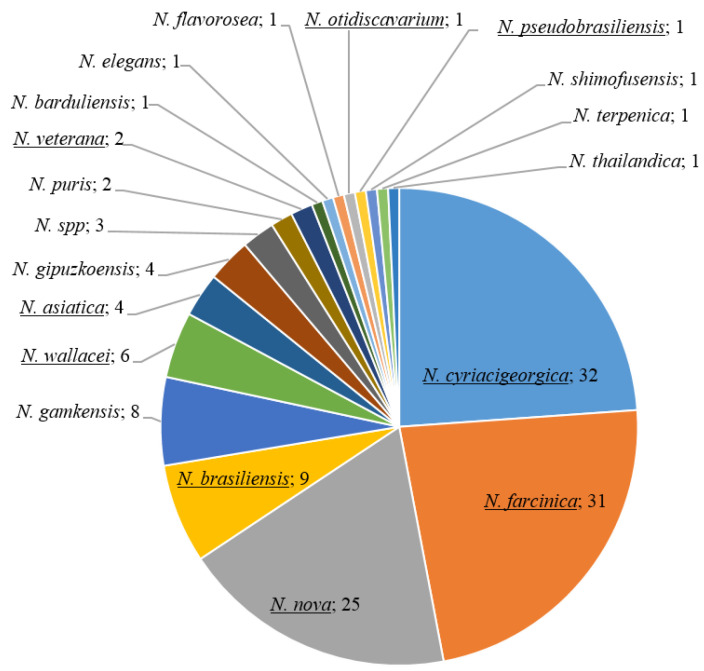
Species identification of 134 *Nocardia* isolates based on the sequencing of 16S rRNA gene and *hsp65* genes. Underlined species were included in the VITEK^®^-MS IVD V3.2 database.

A total of 134 Nocardia isolates were identified by sequencing of 16S rRNA and the hsp65 gene. This collection contained 18 different species, including 9 (111 isolates) contained in the VITEK^®^-MS IVD V3.2 database (Figure 1). A total of three isolates were identified at the genus level by the reference methods: one strain phylogenetically related to *N. brevicatena* and *N. paucivorans*, and two isolates phylogenetically related to *N. gamkensis*, *N. exalbida*, and *N. arthritidis*.

### 2.2. Performance of VITEK^®^-MS for Nocardia Species Identification Using Direct Deposit

VITEK^®^-MS provided an interpretable *Nocardia* species identification (i.e., with a confidence ≥ 99.9%) for 109/134 (81.3%) isolates, and no interpretable identification after 3 runs for 25/134 (18.7%) isolates (Table 1).

### 2.3. Discrepancy Analysis

The overall agreement of *Nocardia* species identification by VITEK^®^-MS compared to 16S rRNA and *hsp65* sequencing-based identification was 78.4% (105/134; 95% confidence interval, CI [70.4; 85.0]) including 1 strain correctly identified at the complex level. This strain, identified as *N. elegans* using gene sequencing-based identification and, therefore, belonging to the *N. nova* complex [14], was found to be *N. africana*/*nova* with VITEK^®^-MS. Taking into account only the species contained in the VITEK^®^-MS IVD V3.2 database, the overall agreement was significantly higher, 93.7% (104/111; 95%CI [87.4; 97.4]).

Of *Nocardia* isolates, 2/3 (67.2%, 90/134) were identified after the first run, of which 53/134 (39.6) were successful in both wells and 37/134 (27.6%) were successful in only one of the two wells (Table 1).

In total, 4 (3.0%) isolates were incorrectly identified by the VITEK^®^-MS IVD V3.2 database (Table 1). One strain was identified as *N. beijingensis* using the VITEK^®^-MS IVD V3.2 database (successful in only one of the two wells after the second run) and *N. gamkensis* by *hsp65* sequencing-based identification. Two isolates were identified as *N. abscessus* by VITEK^®^-MS V3.2 (successful on two wells/two after the first run) and *N. gipuzkoensis* by *hsp65* sequencing-based identification. One strain was identified as *N. neocaledoniensis* by the VITEK^®^-MS IVD V3.2 database (successful in only one of the two wells after the first run) and as *N. thailandica* by 16S rRNA sequencing-based identification.

## 3. Discussion

The MALDI-TOF identification technique has revolutionized clinical bacteriology by providing a rapid and reliable identification on which clinicians can base their treatment decision [8]. This approach seems particularly interesting for *Nocardia*, since the antibiotic susceptibility profile can be deduced from the identification [3].

According to the species contained in the VITEK^®^-MS IVD V3.2 database, the performance of VITEK^®^-MS for *Nocardia* identification by direct deposit using the VITEK^®^-PICKME^TM^ pen and a formic acid-based protein extraction directly onto the bacterial smear was very good, allowing one to rapidly (in a few minutes) and reliably obtain a bacterial identification for 93.7% of isolates by using the same laboratory workflow as that used for conventional bacteria. This yield was comparable to that found with the Bruker Daltonics MALDI-TOF solution using direct smear (94.5%), obtained on a collection of 82 clinical strains [15], or to that that found with VITEK^®^-MS MALDI-TOF using the sample preparation recommended by the manufacturer (95.5%), and obtained on a collection of 90 clinical strains, of which only 3 strains were of a species not contained in the VITEK^®^-MS database [12]. Importantly, the most frequently isolated *Nocardia* species/complexes in France, i.e., *N. farcinica*, *N. abscessus* complex, *N. nova* complex, and *N. cyriacygeorgica* [16], were correctly identified in more than 90% of cases. Moreover, taking into account all species (contained or not in the VITEK^®^-MS IVD V3.2 database), the use of a VITEK^®^-PICKME^TM^ pen to perform direct *Nocardia* deposit with a formic acid-based protein extraction directly onto the bacterial smear resulted in the same yield as that previously found when using the sample preparation recommended by the manufacturer, i.e., 76%, on a large, 312 Nocardia-strain collection, of which 35 strains were of a species not contained in the VITEK^®^-MS database [9].

From a handling point of view, we found that the use of the VITEK^®^-PICKME^TM^ pen was of great help for picking rough *Nocardia* colonies from agar medium, especially for making the thin deposits necessary for good identification by MALDI-TOF. The direct deposit analysis requires the addition of formic acid directly to the deposit in addition to the α-cyano-4-hydroxycinnamic acid (CHCA) matrix solution, to facilitate the destruction of the bacterial wall and extraction of the proteins. Nevertheless, this additional step was much less time-consuming than the sample preparation recommended by the manufacturer and was fully compatible with a classical bacterial identification workflow. Moreover, it is important to note that, as around a quarter of the *Nocardia* isolates were correctly identified in only one of the two wells, the deposit of two wells (rather than one) increased greatly the frequency of correct identification after the first run, thus avoiding the reanalysis time.

Concerning discordant results, we found that the VITEK^®^-MS, exceptionally, provided an interpretable identification for the isolates for species that were not in the database, resulting in few discrepancies. For 3 out of the 4 discordant results, the species identified by VITEK^®^-MS and 16S rRNA/*hsp65* gene sequencing were phylogenetically very close: *N. abscessus* is phylogenetically very close to *N. gipuzkoensis*—a new species recently described, with a 16S rRNA sequence similarity of 100%, but this was described as susceptible to imipenem [17]—and *N. neocaledoniensis* has previously been described as a species closely related to *N. thailandica* [18]. For the fourth discordant result, however, *N. beijingensis* found by VITEK^®^-MS was phylogenetically distant from *N. gamkensis* found by *hsp65* sequencing [19]. It is of note that only in the two cases where *N. gipuzkoensis* was misidentified as *N. abscessus* could this have led to a change in antibiotic management if the latter was considered as resistant. It was considered as resistant in the 2012 version of the M24 standard published by the Clinical and Laboratory Standards Institute [20], but in the latest edition, it is considered as variable (2018) [3], highlighting the need for drug susceptibility testing in all cases for appropriate antibiotic therapy.

Taking into account the results of the present study, and as recommended elsewhere [21], we advise VITEK^®^-MS users carry out identification at the complex level for *N. nova* and for *N. abscessus*, even if the present collection contained few strains of these species. For the *N. nova* complex, the choice of species within the complex will not affect therapeutic management, as all species in the complex share the same antibiotic profile [3]. For the *N. abscessus* complex, as previously discussed, the choice of species may have an impact on the probabilistic antibiotic therapy. However, VITEK^®^-MS does not seem to have sufficient discriminatory power to predict the species within this complex, at the risk of erroneously modifying the probabilistic antibiotic therapy, which in most cases contains imipenem.

In conclusion, a rapid and reliable *Nocardia* identification using direct deposit by MALDI-TOF VITEK^®^-MS is possible by combining the use of the VITEK^®^-PICKME^TM^ pen to easily pick filamentous and dry *Nocardia* colonies in order to obtain fine and uniform deposits, and a formic acid-based protein extractiondirectly onto the bacterial smear. By eliminating the tedious sample preparation steps, this protocol should, therefore, allow the easy integration of *Nocardia* identification by VITEK^®^-MS within the laboratory workflow.

## 4. Materials and Methods

### 4.1. Nocardia Isolates

A collection of 134 *Nocardia* isolates of different geographical origin (Provincial Laboratory for Public Health, Edmonton, AB, Canada; the Karolinska University Hospital, Stockholm, Sweden; and the Infection Control Lab [NHLS] and Department of Clinical Microbiology and Infectious Diseases, Charlotte Maxeke Hospital, Johannesburg, South Africa), collected by the European Committee on Antimicrobial Susceptibility Testing (EUCAST), was used in this study. *Nocardia* isolates were cultured on Columbia agar + 5% sheep’s blood (COS, bioMérieux, Marcy l’Etoile, France) during 48–72 h, in an aerobic atmosphere. Then, isolated colonies were used to perform *Nocardia* identification by both methods: 16S rRNA/hsp65 amplification and sequencing, and VITEK^®^-MS IVD V3.2 database with direct smear using the VITEK^®^-PICKME^TM^ pen.

### 4.2. 16S rRNA/hsp65 Sequencing-Based Nocardia Identification

DNA extraction was performed using QuickExtract^®^ DNA Extraction solution (Lucigen, Middleton, WI, USA) according to the manufacturer’s recommendations. Species identification were obtained by amplification (real-time PCR) and sequencing (Microsynth, Vaulx-en-Velin, France) of a ~600 base pair (bp) fragment of the 16S rRNA gene [22]. A 440 bp fragment of the *hsp65* gene was also amplified and sequenced [7,16] for species identification of *N. abscessus* complex and phylogenetically related species, such as *N. gipuzkoensis*, as well as for identification for 16S rRNA sequencing of *N. gamkensis*, *N. exalbida*, or *N. arthritidis*. The sequences obtained were analyzed using the free Bio Informatic Bacteria Identification (version IV) web-based tool (https://umr5558-proka.univ-lyon1.fr/lebibi/lebibi.cgi, accessed on 1 July 2021), allowing a fully automated approach to the phylogeny of archaea and bacteria. The species identification chosen corresponded to the species that combined the lowest patristic distance (red point) and proximal sequences (nodal distance, in red in the phylogenetic tree). For the discrimination of closely related species (e.g., *N. vulneris*/*N. brasiliensis*, *N. farcinica*/*N. kroppenstedtii*), the sequences were also compared with those stored in GenBank using BLAST alignment software (https://blast.ncbi.nlm.nih.gov/Blast.cgi, accessed on 1 July 2021) to check the sequence similarity with the type strain. Identification at the species level requires 99.6% sequence similarity with the type strain of a single species. If more than 1 sequence in the database has more than 99.6% sequence similarity, then the species identification with the greatest percentage identity was chosen. If no species combined the lowest patristic distance and proximal sequence, and if species identification in the GenBank database was <99.6%, identification was limited to the genus *Nocardia*.

### 4.3. VITEK^®^-MS IVD v3.2 Database Nocardia Identification

Directly from *Nocardia* 48–72 h culture on COS, colonies were picked and deposited using the VITEK^®^-PICKME^TM^ pen on 2 wells of a single-use target slide (=1 run). Next, the deposits were overlaid with 0.5 μL of formic acid (bioMérieux) and were allowed to dry; they were then overlaid with 1 μL of CHCA matrix solution (bioMérieux) and were allowed to dry again. The *Escherichia coli* reference strain ATCC 8739 was used on each plate for instrument calibration according to the manufacturer’s instructions. Finally, the slide was loaded into the VITEK^®^-MS instrument. The mass spectra obtained were compared with the VITEK^®^-MS IVD V3.2 database according to the manufacturer’s recommended settings. The software produced an identification associated with a confidence level. An identification was considered as interpretable when the confidence level was ≥99.9% on 1 or 2/2 wells. In case of failure, another run of 2 wells was deposited using the same interpretation criteria. After 3 failed runs, the strain was considered as unidentifiable (no identification) by VITEK^®^-MS IVD.

## Figures and Tables

**Table 1:** Accuracy of *Nocardia* identification between VITEK-MS V3.2 results and identification based on 16S rRNA and *hsp65* gene sequencing (reference identification).

Reference Identification	Number (%) of Isolates							
	Correct to Species Level				Correct to Complex Level		Incorrect	No. Identification
	2 Wells/2 after the First Run	1 Wells/2 after the First Run	>First Run (New Deposit)	Total	1 Wells/2 after the First Run	Total		
*N. asiatica* (*n* = 4)								4 (100.0)
*N. barduliensis* ^N^ (*n* = 1)								1 (100.0)
*N. brasilliensis* (*n* = 9)	4 (44.4)	5 (55.6)		9 (100.0)				
*N. cyriacigeorgica* (*n* = 32)	14 (43.7)	10 (31.3)	6 (18.7)	30 (93.7)				2 (6.3)
*N. elegans* ^N^ (*n* = 1)					1 (100.0)	1 (100.0)		
*N. farcinica* (*n* = 31)	16 (51.6)	12 (38.7)	2 (6.5)	30 (96.8)				1 (3.2)
*N. flavorosea* ^N^ (*n* = 1)								1 (100.0)
*N. gamkensis* ^N^ (*n* = 8)							1 (12.5)	7 (87.5)
*N. gipuzkoensis* ^N^ (*n* = 4)							2 (50.0)	2 (50.0)
*N. nova* (*n* = 25)	15 (60.0)	7 (28.0)	3 (12.0)	25 (100.0)				
*N. otitidiscavarium* (*n* = 1)	1 (100.0)			1 (100.0)				
*N. pseudobrasiliensis* (*n* = 1)	1 (100.0)			1 (100.0)				
*N. puris* ^N^ (*n* = 2)								2 (100.0)
*N. shimofusensis* ^N^ (*n* = 1)								1 (100.0)
*N.* spp ^N^* (*n* = 3)								3 (100.0)
*N. terpenica* ^N^ (*n* = 1)								1 (100.0)
*N. thailandica* ^N^ (*n* = 1)							1 (100.0)	
*N. veterana* (*n* = 2)	1 (50.0)		1 (50.0)	2 (100.0)				
*N. wallacei* (*n* = 6)	1 (16.7)	3 (50.0)	2 (33.3)	6 (100.0)				
Total (*n* = 134)	53 (39.6)	37 (27.6)	14 (10.4)	104 (77.6)	1 (0.7)	1 (0.7)	4 (3.0)	25 (18.7)

^N^ indicates species not included in the VITEK^®^-MS IVD V3.2 database; * one strain closest to *N. brevicatena*/*N. paucivorans*; two isolates closest to *N gamkensis*/*N. exalbida*/*N. arthritidis*.

## Data Availability

The data presented in this study are available on request from the corresponding author.
